# Callous‐Unemotional Traits and Aggression: Maternal Parenting as a Developmental Moderator

**DOI:** 10.1002/ab.70087

**Published:** 2026-10-25

**Authors:** Nicholas D. Thomson, Jessica J. James, Abriana M. Gresham, Sophie L. Kjærvik, Brooklyn A. Worthen, Kostas A. Fanti

**Affiliations:** ^1^ Department of Social and Behavioral Sciences Virginia Commonwealth University Richmond Virginia USA; ^2^ Center for Gun Violence Research Virginia Commonwealth University Richmond Virginia USA; ^3^ Department of Humanities and Social Sciences New Jersey Institute of Technology Newark New Jersy USA; ^4^ Norwegian Centre for Research on Violence and Traumatic Stress Oslo Norway; ^5^ Department of Psychology University of Cyprus Nicosia Cyprus

**Keywords:** aggression, callous‐unemotional traits, conduct disorder, parenting

## Abstract

Conduct Disorder (CD) is associated with persistent and severe forms of aggression, particularly among youth with callous‐unemotional (CU) traits. Although CU traits are linked to proactive aggression, less is known about how environmental factors, such as parenting, shape these associations over time. The present study examined whether maternal and paternal warmth and hostility moderated the longitudinal associations between CU traits and proactive and reactive aggression in adolescents with CD. Participants included 148 youth with CD aged 12–17 years (*M*_*age*_ = 15.57, 69% male, 51% African American) assessed at baseline and 6 months. Hierarchical regression models tested moderation effects of maternal and paternal warmth and hostility between CU traits and aggression subtypes while controlling for baseline aggression. Maternal warmth and hostility significantly moderated the association between CU traits and proactive aggression, such that CU traits predicted increases in proactive aggression under conditions of low maternal warmth and high maternal hostility. No significant moderating effects were observed for reactive aggression or paternal parenting. Findings suggest that the influence of CU traits on aggression is context‐dependent and varies across aggression subtypes, with maternal parenting playing a key role in shaping the development of proactive aggression. By highlighting the interaction between dispositional traits and caregiving environments, these results refine theoretical models of aggression and identify potential targets for intervention, suggesting that efforts to increase maternal warmth and reduce hostility may help mitigate risk for severe, goal‐directed aggression in this high‐risk population.

1

Conduct disorder (CD) is a relatively common and impairing childhood psychiatric condition, affecting an estimated 3% of youth worldwide (Erskine et al. 2014; Thomson et al. 2024). It is characterized by persistent patterns of behavior that violate social norms and others’ rights, including aggression, deceitfulness, and rule‐breaking (American Psychiatric Association 2022). Youth with CD are at heightened risk for chronic antisocial behavior, substance misuse, and justice system involvement later in life (Fairchild et al. 2019). Given its chronicity and resistance to intervention (Hawes and Dadds 2021; McDonald et al. 2021), identifying mechanisms that shape the development and maintenance of CD is critical for theory and intervention.

Callous‐Unemotional (CU) traits, characterized by callousness, low empathy, diminished guilt, and shallow affect, identify a particularly high‐risk subgroup of youth with CD (Frick et al. 2014). CU traits are associated with more severe, persistent, and treatment‐resistant forms of aggression (Perlstein et al. 2023; Robertson et al. 2020). Youth with CU traits are particularly prone to engaging in proactive aggression (i.e., deliberate, goal‐directed behavior used to obtain desired outcomes), which is thought to be reinforced through reward‐based learning processes (Blair 2016 2022; Thomson et al. 2024). Compared to youth with CD alone, those with high CU traits exhibit higher levels of proactive aggression and tend to experience lower parental warmth and higher hostility (Frick et al. 2014; Hyde et al. 2016; Waller et al. 2018). However, youth with CU traits also exhibit reactive aggression, which is impulsive and driven by perceived threat (Blair 2016; Thomson et al. 2024), suggesting that CU‐related risk may operate across multiple aggression subtypes (Thomson and Centifanti 2018; Vaughan et al. 2024).

Although existing models emphasize either dispositional risk (e.g., CU traits) or environmental reinforcement through social learning (e.g., Bandura 1973), they do not clearly specify how these processes interact over time in relation to distinct forms of aggression. This gap is particularly important given evidence that parenting practices, such as warmth and hostility, play a central role in socioemotional development (Labella and Masten 2018). Parental warmth, characterized by support and affection, is generally protective, whereas parental hostility is associated with adverse behavioral outcomes, including increased aggression (Garcia et al. 2020; Pinquart 2017). However, it remains unclear whether these parenting behaviors amplify or buffer the effects of CU traits on aggression, and whether such effects differ across proactive and reactive aggression. Further, although maternal hostility may be a stronger predictor of negative outcomes than paternal hostility (Khaleque 2017), less is known about whether similar patterns emerge for parental warmth (Vaughan et al. 2021).

The present study addresses these gaps by examining whether maternal and paternal warmth and hostility moderate the longitudinal associations between CU traits and proactive and reactive aggression in a high‐risk sample of youth with CD. By integrating dispositional (i.e., CU traits) and environmental (i.e., parenting) variables and differentiating between aggression subtypes, this study aims to refine theoretical models of aggression by clarifying how individual vulnerabilities and caregiving environments jointly shape the development of aggressive behavior over time. We hypothesized that CU traits would predict increases in both proactive and reactive aggression, with stronger effects for proactive aggression. Consistent with social learning theory (Bandura 1977), we expected that parental hostility would strengthen and parental warmth would weaken the association between CU traits and aggression.

## 2 Methods

### 2.1 Participants and Procedure

Participants were part of a larger longitudinal study and were recruited through a large healthcare network in Virginia. Participants were 148 adolescents (M_age_ = 15.57, SD = 1.35) diagnosed with CD. Most participants were male (69%) and identified as African American (51%), White (42%), mixed race (4%), or other (3%). Prior to study participation, youth provided assent, and their caregivers provided consent in separate and private testing rooms. Data were collected at baseline (T1) and 6‐month follow‐up (T2). Participants received $200 for participating in the larger study, examining the stability of CU traits among youth with CD (R01MH123535; PI: Thomson).

## 3 Measures

### 3.1 Callous‐Unemotional Traits (T1)

CU traits were assessed using the Inventory of Callous‐Unemotional Traits (ICU; Kimonis et al. 2008). Internal consistency was acceptable, *α* = 0.81.

### 3.2 Parenting (T2)

Maternal and paternal warmth and hostility were assessed using the Quality of Parental Relationships Inventory (Crick and Dodge 1994). Internal consistency was high across subscales, αs = 0.89−0.97.

### 3.3 Aggression (T1, T2)

Proactive and Reactive aggression were measured using the Reactive–Proactive Aggression Questionnaire (RPQ; Raine et al. 2006). Reliability was strong across time points, αs = 0.70−0.90. Further descriptive information about each measure is reported in Supplemental S1.

## 4 Analytic Plan

Hierarchical regression models tested whether parental warmth and hostility moderated associations between CU traits (T1) and aggression (T2), controlling for baseline aggression, age, and sex. One participant was excluded from the reactive aggression analysis due to missing complete data. A detailed data analytic plan is provided in Supplemental S2.

## 5 Results

### 5.1 Preliminary Analysis

CU traits were positively associated with proactive aggression at T1, *r* = 0.27, *p* = 0.001, and at T2, *r* = 0.24, *p* = 0.004, but were not significantly associated with reactive aggression at T1 or T2, *ps* = 0.12 − 0.13. Proactive and reactive aggression were positively associated at T1, *r* = 0.57, *p* < 0.001, and T2, *r* = 0.59, *p* < 0.001, and showed moderate stability across time, *r*s = 0.42 − 0.47. *p*s < 0.001. Maternal warmth was negatively associated with both proactive, *r* = −0.23, *p* = 0.006, and reactive, *r* = −0.28, *p* < 0.001, aggression at T2, whereas maternal hostility was positively associated with proactive, *r* = 0.42, *p* < 0.001, and reactive, *r* = 0.35, *p* < 0.001, aggression at T2. Paternal warmth was negatively associated with proactive, *r* = −0.18, *p* = 0.032, and reactive, *r* = −0.18, *p* = 0.032, aggression at T2 (see Table 1).

**Table 1:** Descriptive statistics and correlations among study variables.

	*M*	SD	1	2	3	4	5	6	7	8	9	10	11
1. Age	15.57	1.35	—										
2. Sex	0.69	0.47	−0.02	—									
3. CU Traits	23.90	8.18	0.10	0.12	—								
4. Reactive Aggression (T1)	8.90	4.66	0.03	−0.10	0.12	—							
5. Proactive Aggression (T1)	1.84	2.43	−0.06	0.00	0.27**	0.57***	—						
6. Reactive Aggression (T2)	7.55	4.68	−0.02	−0.12	0.13	0.47***	0.27**	—					
7. Proactive Aggression (T2)	1.99	2.95	0.01	−0.02	0.24**	0.31***	0.42***	0.59***	—				
8. Maternal warmth (T2)	20.76	6.69	0.05	0.08	−0.06	−0.13	−0.13	−0.28***	−0.23**	—			
9. Paternal warmth (T2)	15.97	8.99	0.04	0.20*	−0.11	−0.09	−0.12	−0.18*	−0.18*	0.43***	—		
10. Maternal hostility (T2)	6.21	5.61	−0.05	−0.07	0.04	0.16	0.18*	0.35***	0.42***	−0.47***	−0.08	—	
11. Paternal hostility (T2)	4.00	5.41	−0.04	0.04	0.06	0.18*	0.24**	0.10	0.13	−0.07	−0.17*	.15	—

Abbreviations: T1 = baseline, T2 = 6 months, CU = callous‐unemotional traits.

* *p* < 0.05

** *p* < 0.01

*** *p* < 0.001.

## 6 Associations Between CU Traits, Parenting, and Aggression

### 6.1 Proactive Aggression

Baseline proactive aggression and maternal hostility, *ps* < 0.001, significantly predicted proactive aggression at T2. The addition of the maternal and paternal warmth interaction terms significantly improved the model, Δ*R* ^2^ = 0.095, *F*(2, 137) = 12.12, *p* < 0.001, with a significant CU traits × maternal warmth interaction, *p* < 0.001. The addition of the maternal and paternal hostility interaction terms also significantly improved the model, Δ*R* ^2^ = 0.036, *F*(2, 137) = 4.16, *p* = 0.018, with a significant CU traits × maternal hostility interaction, *p* = 0.005 (see Table 2).

**Table 2:** Hierarchical Regression Predicting Proactive Aggression.

	Parental warmth				Parental hostility			
Predictors	*B*	*SE*	*β*	*R* ^*2*^	*B*	*SE*	*β*	*R* ^*2*^
Step 1				0.37***				0.37***
Age	−0.02	0.15	−0.01		−0.02	0.15	−0.01	
Sex	0.10	0.43	0.02		0.04	0.46	0.01	
Baseline aggression	0.46***	0.09	0.37***		0.46***	0.09	0.37***	
CU traits	0.05*	0.02	0.14*		0.05	0.03	0.13	
Maternal warmth	−0.04	0.33	−0.01		−0.09	0.36	−0.02	
Paternal warmth	−0.11	0.23	−0.03		−0.18	0.24	−0.06	
Maternal hostility	2.79***	0.49	0.35***		2.32***	0.51	0.34***	
Paternal hostility	−0.28	0.46	−0.04		−0.28	0.46	−0.04	
Step 2				0.47***				0.41***
CU traits × Maternal warmth	−0.18***	0.04	−0.34***					
CU traits × Paternal warmth	0.02	0.03	0.05					
CU traits × Maternal hostility					0.15**	0.05	0.20**	
CU traits × Paternal hostility					−0.06	0.06	−0.07	

*Note: N* = 148.

Abbreviation: CU = callous‐unemotional traits.

* *p* < 0.05

** *p* < 0.01

*** *p* < 0.001.

Simple slopes analyses indicated that CU traits predicted increases in proactive aggression under conditions of low maternal warmth, *b* = 0.20, *SE* = 0.04, *t* = 5.01, *p* < 0.001, but not high maternal warmth, *b* = −0.07, *SE* = 0.04, *t* = −1.85, *p* = 0.066. Similarly, CU traits predicted increases in proactive aggression under high maternal hostility, *b* = 0.12, *SE* = 0.04, *t* = 3.32, *p* = 0.001, but not low maternal hostility, *b* = −0.02, *SE* = 0.04, *t* = −0.63, *p* = 0.530. No significant moderating effects were observed for paternal parenting, *p*s > 0.05 (See Figures 1 and 2).

**Figure 1:**
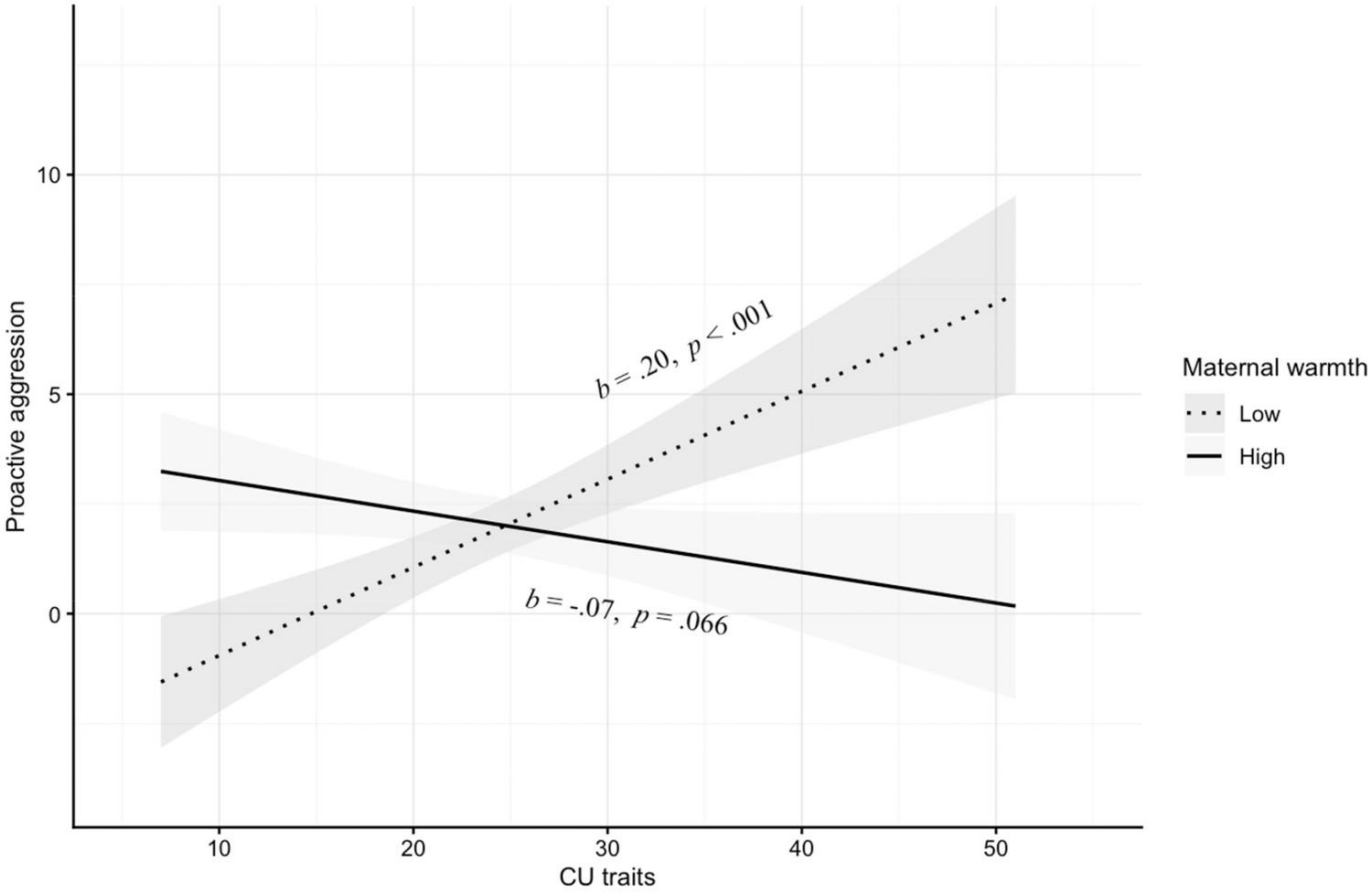
Maternal warmth as a moderator between CU traits and proactive aggression at 6 months. *Note:* The shaded area represents 95% confidence intervals.

**Figure 2:**
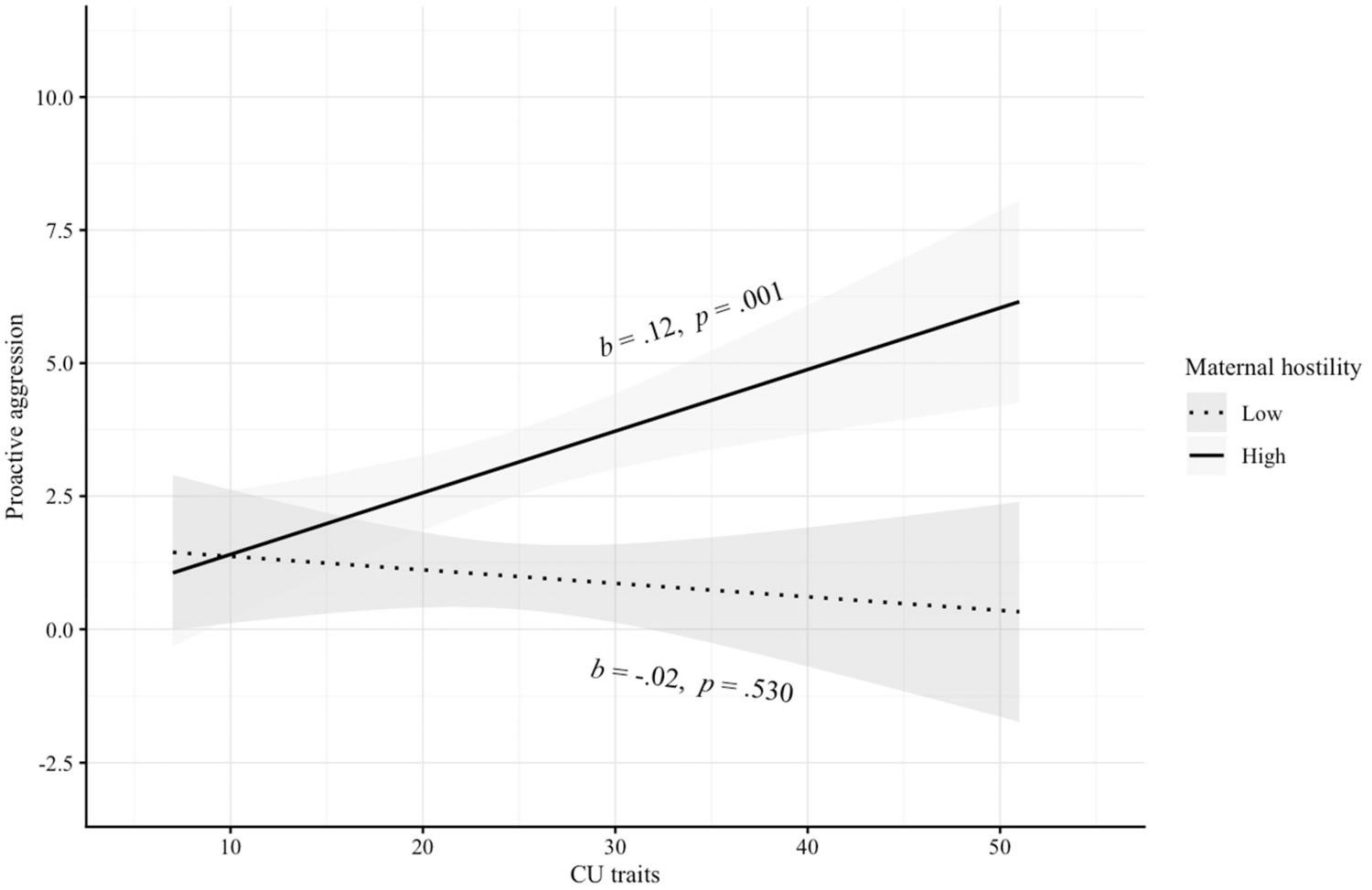
Maternal hostility as a moderator between CU traits and proactive aggression at 6 months. *Note:* The shaded area represents 95% confidence intervals.

### 6.2 Reactive Aggression

Baseline reactive aggression, *p* < 0.001, and maternal hostility, *p* = 0.004, predicted reactive aggression at T2. However, no significant interactions emerged between CU traits and parenting variables, *p*s > 0.05, indicating that parenting did not moderate associations between CU traits and reactive aggression (See Table 3).

**Table 3:** Hierarchical regression predicting reactive aggression.

	Parental warmth				Parental hostility			
Predictors	*B*	SE	*β*	*R* ^*2*^	*B*	SE	*β*	*R* ^*2*^
Step 1				0.32				0.32
Age	−0.01	0.25	−0.00		−0.01	0.25	−0.00	
Sex	−0.52	0.74	−0.05		−0.52	0.74	−0.05	
Baseline aggression	0.40***	0.07	0.39***		0.40***	0.07	0.39***	
CU traits	0.03	0.04	0.06		0.03	0.04	0.06	
Maternal warmth	−0.54	0.57	−0.09		−0.54	0.57	−0.09	
Paternal warmth	−0.42	0.39	−0.09		−0.42	0.39	−0.09	
Maternal hostility	2.46**	0.84	0.24**		2.46**	0.84	0.24**	
Paternal hostility	−0.14	0.77	−0.01		−0.14	0.77	−0.01	
Step 2				0.35				0.34
CU traits × Maternal warmth	−0.14	0.07	−0.17					
CU traits × Paternal warmth	0.01	0.04	0.02					
CU traits × Maternal hostility					0.11	0.09	0.09	
CU traits × Paternal hostility					0.15	0.10	0.11	

*Note: N* = 147.

Abbreviation: CU = callous‐unemotional traits.

* *p* < 0.05

** *p* < 0.01

*** *p* < 0.001.

## 7 Discussion

The present study examined how parental warmth and hostility moderated the longitudinal associations between CU traits and proactive and reactive aggression in youth with CD. By integrating dispositional and environmental variables, these findings extend current models of aggression, which have typically emphasized either trait‐based risk or reinforcement processes (Bandura 1977; Blair 2016, 2022) but have rarely examined how these processes interact over time to influence aggression subtypes (Labella and Masten 2018). Our findings suggest that parenting behaviors may influence the strength of the association between CU traits and changes in proactive aggression over time.

Consistent with prior research, CU traits were associated with higher levels of aggression, particularly proactive aggression (Frick et al. 2014; Thomson and Centifanti 2018). This pattern supports theoretical accounts suggesting that emotional deficits characteristic of CU traits reduce internal inhibitions against harming others and facilitate goal‐directed aggression (Blair 2016; Frick et al. 2014). The stronger association with proactive aggression aligns with models proposing that CU traits are particularly linked to instrumental, reward‐driven forms of aggression (Blair 2016, 2022; Thomson and Centifanti 2018).

Critically, these findings demonstrate that these associations are context‐dependent. Although broadly consistent with prior research, these findings extend the literature by demonstrating these associations in a high‐risk sample of youth with CD, a population characterized by elevated aggression and psychosocial risk. Maternal warmth and hostility moderated the relationship between CU traits and proactive aggression, such that CU traits predicted increases in proactive aggression only under conditions of low maternal warmth and high hostility. These results suggest that environmental inputs can amplify or attenuate dispositional risk and are consistent with developmental models emphasizing the role of caregiving environments in shaping behavioral outcomes (Labella and Masten 2018) and behavioral genetic research demonstrating that the expression of CU traits is contingent on environmental context, particularly parenting (Hyde et al. 2016; Tomlinson et al. 2025; Waller et al. 2018).

From a theoretical perspective, these findings help bridge dispositional and social learning accounts of aggression. While CU traits may predispose youth toward proactive aggression, hostile caregiving environments may solidify and normalize such behavior through modeled interactions and reinforcement processes (Bandura 1977; Liu et al. 2022; Thomas et al. 2018), increasing the likelihood over time. Conversely, warm parenting may disrupt this pathway. These results support an integrative model in which aggression emerges from the interaction between individual traits and environmental contexts.

Importantly, these moderating effects were specific to proactive aggression. Parenting did not moderate the associations between CU traits and reactive aggression, suggesting that reactive aggression may be more strongly driven by other factors beyond those examined in this study, such as emotion dysregulation and threat sensitivity (Crick and Dodge 1994; Dodge and Coie 1987; Thomson et al. 2021 2024). This distinction highlights the importance of differentiating aggression subtypes in theoretical models.

Maternal, but not paternal, parenting emerged as a significant moderator, suggesting that associations with caregiving may not operate uniformly across caregivers. This finding is consistent with prior work indicating that maternal parenting may be more strongly associated with youths’ socioemotional development and behavioral outcomes (Khaleque 2017; Thomson et al. 2020).

## 8 Implications

Our findings suggest that theoretical models of aggression should more explicitly incorporate interactions between CU traits and parenting environments, particularly to better understand proactive aggression. Interventions that increase parental warmth and reduce hostility may be especially effective in disrupting pathways between CU traits and severe aggression, consistent with evidence supporting parent‐focused interventions to reduce youth aggression (Perlstein et al. 2023).

## 9 Limitations

The present study has several limitations. First, measures relied on youth self‐reports, which may introduce shared method variance and potential biases, particularly in assessing CU traits (Kimonis et al. 2008). Future research would benefit from incorporating multi‐informant approaches, including caregiver and teacher reports, to provide a more comprehensive assessment of behavior (Frick et al. 2014). Second, although the study employed a longitudinal design, the 6‐month follow‐up period may be insufficient to capture longer‐term developmental changes in aggression, offering only a preliminary view of developmental trajectories (Fanti et al. 2013). Third, the sample was drawn from a specific region of the United States, which may limit generalizability. Cultural and contextual factors play an important role in shaping parenting practices and the expression of aggression (Fuentes et al. 2022; Liu et al. 2022), and perceptions of warmth and hostility may vary across cultural contexts (Khaleque 2017), with implications for the development of aggressive behavior (Labella and Masten 2018). Future research should examine these processes in more diverse and cross‐cultural samples.

## 10 Conclusion

The present study demonstrates that the influence of CU traits on aggression is conditional on caregiving context and varies across aggression subtypes. By highlighting the interaction between dispositional traits and parenting environments, these findings refine theoretical models of aggression and identify potential targets for intervention.

## Ethics Statement

All procedures performed in studies involving human participants were performed with approval from a relevant institutional review board.

## Conflicts of Interest

The authors declare no conflicts of interest.

## Supporting information


Supporting File

## Data Availability

Data and materials may be made available upon request from the corresponding author. Data are not publicly available due to the potential for personal identification of participants in a sensitive population.
