# Supplementary material for: Callous‐Unemotional Traits and Aggression: Maternal Parenting as a Developmental Moderator
Source: Aggress Behav. 2026 Oct 25;52(6):e70087. doi: 10.1002/ab.70087 (PMC13696014; doi:10.1002/ab.70087)
Supplement: Supplementary file 1 [file AB-52-e70087-s001.docx]

**Supplementary Materials**

**S1. Measures**

**Callous–Unemotional Traits (ICU)**

CU traits were assessed at baseline (T1) using the Inventory of Callous–Unemotional Traits (ICU; Kimonis et al., 2008), a 24-item self-report measure. Items are rated on a 3-point scale (1 = not at all true to 3 = definitely true). The measure includes both positively and negatively worded items; negatively worded items were reverse-coded prior to scoring so that higher scores reflect greater CU traits. Internal consistency in the current sample was acceptable (α = .81).

**Parenting: Warmth and Hostility (QPRI)**

Parenting behaviors were assessed at follow-up (T2) using the Quality of Parental Relationships Inventory (QPRI; Conger et al., 1994), a 42-item self-report measure assessing adolescents’ perception of caregiver behaviors. Participants reported on their experience with maternal and paternal caregivers. The QPRI specifies that the assessment pertains to the time since last assessment (i.e., the last 6 months); therefore, responses were interpreted as reflecting adolescents’ perceptions of their current and recent caregiving environment.

Subscales included:

- Maternal warmth (9 items)
- Maternal hostility (12 items)
- Paternal warmth (9 items)
- Paternal hostility (12 items)

Items were rated on a 4-point scale (0 = always to 3 = never) and coded such that higher scores reflected greater warmth or hostility. Adolescents without a maternal or paternal caregiver were excluded from the analyses because all models included both maternal and paternal parenting variables.

Internal consistency was high across subscales:

- Maternal warmth (α = .95)
- Maternal hostility (α = .89)
- Paternal warmth (α = .97)
- Paternal hostility (α = .92)

**Reactive and Proactive Aggression (RPQ)**

Aggression was assessed at both baseline (T1) and follow-up (T2) using the Reactive–Proactive Aggression Questionnaire (RPQ; Raine et al., 2006), a 23-item self-report measure.

The RPQ includes:

- Reactive aggression (11 items; e.g., aggression in response to provocation)
- Proactive aggression (12 items; e.g., goal-directed aggression)

Items are rated on a 3-point scale (0 = never, 1 = sometimes, 2 = often). Subscale scores are computed by summing relevant items.

Internal consistency was strong across timepoints:

- T1 reactive α = .84; proactive α = .70
- T2 reactive α = .88; proactive α = .81

**S2. Data Analytics Plan**

To examine changes in aggression over time, baseline (T1) aggression was included as a covariate, and follow-up (T2) aggression served as the primary outcome. CU traits were assessed at T1 and treated as a stable predictor. Parenting variables were assessed at T2 and examined as moderators of the association between baseline CU traits and follow-up aggression. Because parenting was measured only at T2, the findings should be interpreted as reflecting associations between perceived parenting and changes in aggression over the study period, rather than the prospective effect of parenting on aggression.

Pearson correlations were conducted to examine associations among CU traits, aggression, and parenting variables. Complete-case analyses were conducted separately for each regression model. One participant was excluded from reactive aggression models due to missing data. Moderation analyses were conducted using hierarchical regression models in R (R Core Team, 2020). Separate models were estimated for reactive and proactive aggression. Each model included:

- Step 1: Age, sex, baseline aggression, CU traits, and the main effects of maternal warmth, paternal warmth, maternal hostility, and paternal hostility.
- Step 2: For warmth models, CU traits × maternal warmth and CU traits × paternal warmth interaction terms were added. For hostility models, CU traits × maternal hostility and CU traits × paternal hostility interaction terms were added.

**Separate warmth and hostility models were retained because of power considerations associated with the number of predictors in a combined model. A sensitivity analysis indicated that a combined model including all warmth and hostility main effects and interaction terms (12 predictors) would have 80% power to detect an incremental effect of Δ*R*² ≥ .084 or greater. This was considered important because the observed incremental effect for the hostility model was smaller (Δ*R*² = .036). Separate models therefore provided greater sensitivity to potentially smaller interaction effects while retaining both maternal and paternal parenting variables within each model.**

Significant interactions were probed using simple slopes analyses.
